# Enhanced Diagnosis of Generalized Pustular Psoriasis With the Legit.Health Device as a Diagnosis Support Tool: Multireader Multicase Study

**DOI:** 10.2196/82030

**Published:** 2026-06-10

**Authors:** Alfonso Medela, Ignacio Hernández Montilla, Alberto Sabater, Andy Aguilar, Taig Mac Carthy, Gurpreet Singh Chowdhry, Juan Semeco, Antonio Martorell

**Affiliations:** 1 Department of Medical Data Science, Legit.Health, Gran Via 1 BAT Tower, Bilbao, 48001, Spain, 34 638127476; 2 Department of Clinical Endpoint Innovation, Legit.Health, Bilbao, Spain; 3 TA Inflammation, Boehringer Ingelheim International GmbH, Ingelheim am Rhein, Germany; 4 TA Inflammation Medicine, Boehringer Ingelheim International GmbH, Ingelheim am Rhein, Germany; 5 Dermatology Department, Hospital de Manises, Valencia, Spain

**Keywords:** generalized pustular psoriasis, psoriasis, hidradenitis suppurativa, artificial intelligence, AI, rare diseases, diagnostic accuracy

## Abstract

**Background:**

Generalized pustular psoriasis (GPP) is a rare, chronic, systemic inflammatory disease with an unpredictable and heterogeneous clinical course characterized by chronic symptoms and periods of flaring. GPP presents diagnostic challenges due to its rarity and high similarity to other dermatologic diseases.

**Objective:**

This study aimed to assess the performance of Legit.Health, a medical device powered by artificial intelligence software, in assisting health care practitioners (HCPs) in identifying GPP.

**Methods:**

The medical device used in this study includes a deep neural network for skin disease recognition trained on thousands of images of over 200 skin conditions (classified based on the *International Classification of Diseases, 11th Revision*). The sensitivity and specificity of the algorithm for the differential diagnosis of GPP were assessed. Due to the scarcity of GPP-related images, the medical device was fine-tuned using a dataset that included 4397 new GPP images. Thereafter, 15 HCPs (n=11, 73.3% primary care practitioners and n=4, 26.7% dermatologists) prospectively reviewed a total of 100 images of 15 visually similar skin conditions virtually in a clinical setting. After their diagnostic prediction, Legit.Health provided a prompt giving them the top 5 possible skin conditions to assist them with their choice.

**Results:**

Legit.Health demonstrated high accuracy in identifying GPP, with top-1, top-3, and top-5 sensitivity and specificity of 0.80, 0.86, and 0.90 and 0.99, 0.99, and 0.96, respectively. Results showed a notable increase in the diagnostic accuracy of the HCPs with assistance from Legit.Health (*P<*.001), with an increase in GPP diagnostic accuracy of 22.97% overall, 24.24% for primary care practitioners, and 19.45% for dermatologists.

**Conclusions:**

This improvement highlights the potential of Legit.Health in assisting HCPs in diagnosing rare diseases such as GPP, particularly in primary care settings where expertise may be limited, thereby improving patient outcomes.

## Introduction

Generalized pustular psoriasis (GPP) is a rare, systemic neutrophilic inflammatory disease with an unpredictable and heterogeneous clinical course [1-5], which is characterized by chronic symptoms such as pustules, erythema, and periods of flaring [5,6]. GPP is associated with a considerable clinical burden, which can greatly impact patients’ lives both physically and emotionally. Patients with GPP may experience multiple flares per year that can be triggered by infections, stress, medication withdrawal (such as from corticosteroids and biologics), menstruation, and pregnancy [5,7-12]. GPP flares and symptoms are often unpredictable and highly heterogeneous due to the presence of cyclical variations in disease severity that occur over long periods [13,14]. GPP manifests as painful, itchy, and visible pustules and also induces systemic symptoms, including fever and fatigue [5]. Without timely and appropriate intervention, GPP flares can lead to life-threatening complications that require emergency treatment, such as multisystem organ failure and sepsis [5,6]; therefore, there is a critical need for prompt diagnosis and effective management of symptoms.

A study by Reisner et al [15] of 66 patients living with GPP in the United States showed that 86% experienced 2 or more flares annually. Despite the profound impact of GPP on patients, the diagnostic process can be challenging. Insights from the study by Reisner et al [15] highlighted significant delays and misdiagnoses experienced by patients. Approximately 40% of surveyed patients reported that it took years for them to receive an accurate diagnosis of GPP, with over 50% being consulted by multiple health care practitioners (HCPs) before the correct diagnosis was made [15]. A common reason for delays in diagnosis is the frequent misdiagnosis of GPP as an infection, leading to incorrect treatments and delayed specialist referrals [16]. This misdiagnosis is attributed to the rarity of GPP and the consequent lack of experience and knowledge of the presentation and pathogenesis of GPP among many HCPs [16].

The impact of GPP extends beyond physical symptoms as GPP can have a profound effect on patients’ emotional health and quality of life, influencing daily activities, work, and social relationships [15]. The patients in the study by Reisner et al [15] reported a significant emotional impact, with 71% reporting that they lived in fear of flares and two-thirds reporting that they experienced anxiety related to their GPP. Emotional stress, which is itself a major trigger for flares, affected 83% of those surveyed [15].

In recent years, artificial intelligence (AI) has been increasingly implemented in the medical field, offering promising advancements in disease diagnosis and management. However, despite the increased number of AI algorithms, their implementation in clinical practice remains challenging. Many AI tools lack the appropriate medical device certification and fail to prove their usefulness in real-world settings. Therefore, there is a pressing need for AI solutions that are technically proficient and beneficial in real-world clinical practice.

Legit.Health, a medical device powered by AI, uses computer vision algorithms (deep neural networks) that have been shown to be effective in many clinical settings, such as in assessing the severity of hidradenitis suppurativa (HS) [17], urticaria [18], and atopic dermatitis [19]. In this study, we aimed to evaluate the diagnostic accuracy of HCPs for GPP before and after the use of the Legit.Health device and its skin disease recognition algorithm. This multireader, multicase study is the first to evaluate the real-world clinical utility of an AI-powered medical device in improving diagnostic accuracy for GPP, addressing a critical gap in dermatologic care.

## Methods

The study methodology adheres to the CLEAR Derm (Checklist for Evaluation of Image-Based AI Reports in Dermatology) guidelines [20].

### Device Fine-Tuning and Validation

#### Dataset

**Figure 1.**
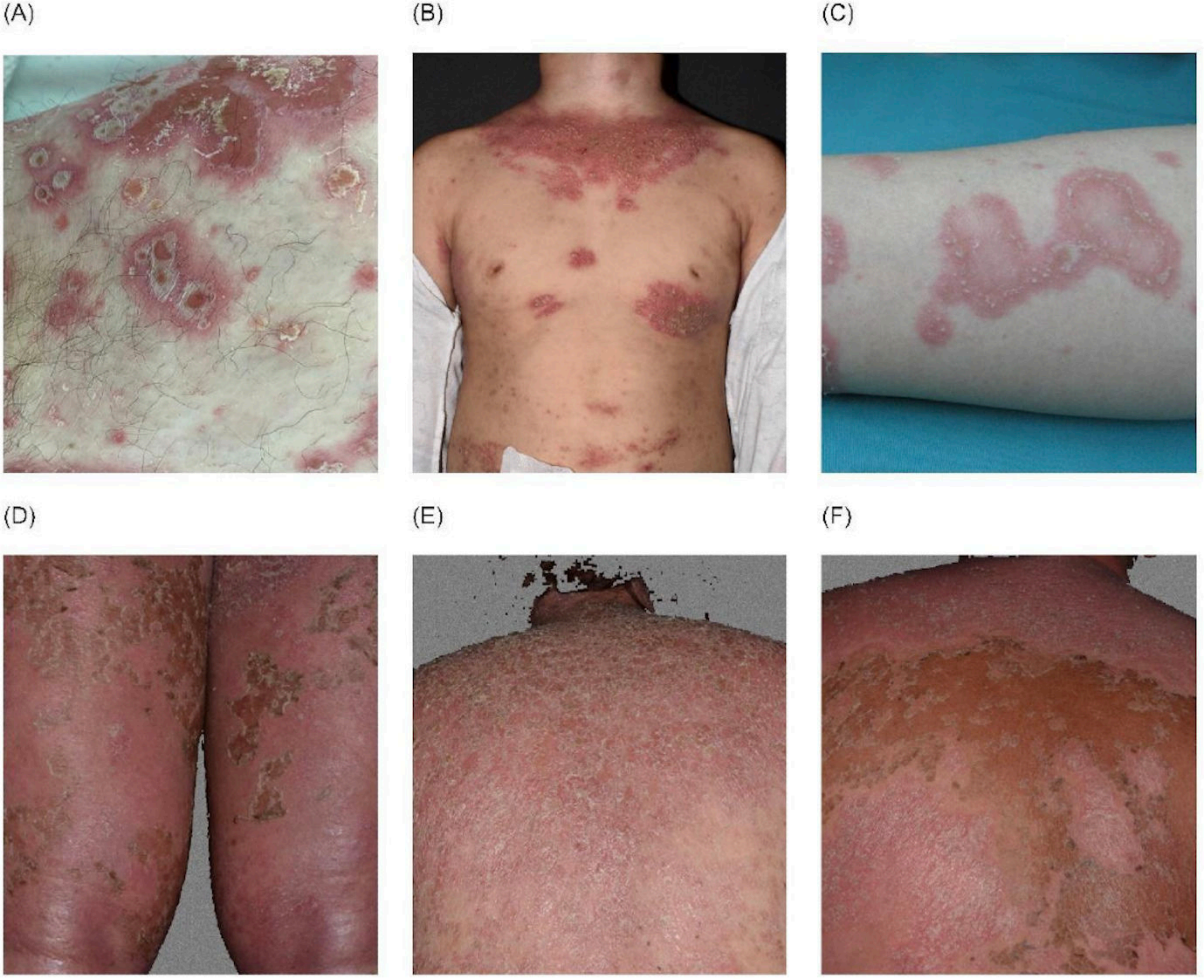
Examples of generalized pustular psoriasis images from the dataset. Images A, B, and C correspond to samples from the first batch, while D, E, and F correspond to samples from the second.

Legit.Health, a class IIb, CE-marked medical device (International Organization for Standardization 13485; approval number: MD792784; certificate number: MDR 792790; single registration number: ES-MF-000025345), among other features, incorporates a neural network for skin disease recognition trained on an extensive dataset, which comprises hundreds of thousands of skin images and represents over 200 categories from the *International Classification of Diseases, 11th Revision* (*ICD-11*). Images from the Legit.Health dataset were acquired using a combination of digital cameras, smartphone cameras, and dermatoscopes. Although this dataset already included a small subset of images of patients with GPP, 2 additional batches of images (Figure 1) were incorporated to enhance the differential diagnosis capabilities for GPP. These 2 batches of patient images were supplied by Boehringer Ingelheim and acquired using digital cameras. The first batch comprised 101 images, most of which focused on close-up views of small and discrete body sites (Figure 1A-C). These images provided detailed views of the pustules, which are characteristic of GPP, facilitating precise lesion recognition and analysis. The second batch comprised 4296 high-resolution images depicting larger body sites (ie, images of the trunk and legs) viewed from both the front and back (Figure 1D-F). This batch of images provided a comprehensive visual context, capturing the widespread distribution of pustules that is typical in GPP. All images were sourced from patients with confirmed GPP diagnoses (see Table 1 for demographic information), ensuring the reliability and accuracy of the dataset. In both batches, images depicting a patient with GPP with no visible signs at the time of the picture were also identified; therefore, no additional review or diagnosis of each image was required. These patients were not actively involved in the testing of the Legit.Health device, and no patient images were presented to the public. Once the new batches of GPP images were provided, they were properly stratified into training, validation, and test sets to prevent data leakage. The training and validation images were then combined with the Legit.Health dataset to refine the algorithm’s response for GPP.

**Table 1.** Summary of patient demographics for the dataset of generalized pustular psoriasis images (N=4397).

Sex	Number of patients	Samples, n (%)
Female	36	3024 (68.8)
Male	21	1373 (31.2)

For this study, we prepared a subset of 100 images representing a range of dermatologic conditions, including GPP and various conditions commonly encountered in primary and secondary care that may resemble GPP and which, thereby, complicate the diagnostic process. The list of conditions included in this study was defined by one of the authors (A Martorell) and aimed to simulate real-world clinical scenarios and test the device’s ability to accurately differentiate GPP from other similar skin conditions. To create this subset, we picked images from the test split of the Legit.Health dataset and the test split of the new GPP image batches. No formal sample size calculation was carried out. This initial dataset was deemed sufficient to answer the research question due to the number of images within the existing neural network for skin disease recognition incorporated into the Legit.Health medical device.

### Fine-Tuning

As indicated above, Legit.Health already included GPP within its diagnostic outputs, classified as EA90.40 according to the *ICD-11* [21]. However, due to the scarcity of GPP cases and related GPP images compared with other *ICD-11* categories, the medical device’s algorithm was fine-tuned using an updated dataset that included the 2 new batches of GPP images. We began by detecting the images that did not present visual signs of GPP (6/101, 5.9% of the images from the first batch and 688/4296, 16.0% of the images from the second batch) and removing them from the dataset’s GPP category. The remaining images (n=3608) were split into training (n=2563), validation (n=546), and test (n=499) sets. As the images included metadata such as patient identification codes, we were able to stratify the dataset and avoid data leakage at every stage. The device’s skin disease recognition algorithm was retrained using the original setup and procedures established by the manufacturer, integrating the new GPP images with the existing validated dataset.

To enhance the performance and generalizability of the medical device, specific data augmentation techniques such as random color jittering, histogram equalization, motion blur, rotation, and resizing were applied to the GPP images. These techniques accounted for variations in image quality, lighting conditions, and background features, thereby increasing the robustness of the medical device. The augmented dataset helped in simulating a diverse range of clinical scenarios, which is crucial for the accurate recognition of GPP in real-world settings.

The training process used superconvergence methods, significantly expediting the learning phase and optimizing the device’s ability to handle the increased volume of data. Superconvergence leverages a cyclic learning rate schedule, which allows the device to converge rapidly while maintaining high accuracy even with the substantial addition of new images [22].

#### Validation

The performance of Legit.Health was evaluated using the top-1, top-3, and top-5 sensitivity and specificity metrics for the GPP category. While top-1 sensitivity and specificity were equivalent to the common sensitivity and specificity metrics, the top-3 and top-5 variants accounted for cases in which the correct GPP category was not exactly in the first position of the device’s output but appeared among the top 3 and top 5 predicted categories. These variants provided a more realistic understanding of the device’s performance; moreover, they provided a comprehensive understanding of the medical device’s accuracy and its ability to correctly identify GPP among other dermatologic conditions.

### Clinical Study

A multireader, multicase clinical study was conducted to assess the effectiveness of Legit.Health in helping enhance diagnostic accuracy for GPP among HCPs. This evaluation involved 15 HCPs (female: n=8, 53.3%) comprising 11 (73.3%) primary care practitioners (PCPs; female: n=6, 54.5%) and 4 (26.7%) dermatologists (female: n=2, 50%), thereby providing insights on the usefulness of the medical device across different levels of dermatologic expertise.

### Participant Selection and Training

The HCPs (all based in Spain) who participated in this study were selected based on their clinical experience and willingness to commit to the full evaluation process. Prior to the evaluation, the HCPs received training on how to use Legit.Health. This included an overview of the device’s diagnostic capabilities, guidance on interpreting its output, and an understanding of the confidence metrics provided by the system. They were also given a brief introduction to the evaluation experiment without any hints of which conditions they would be presented with.

### Design

**Figure 2.**
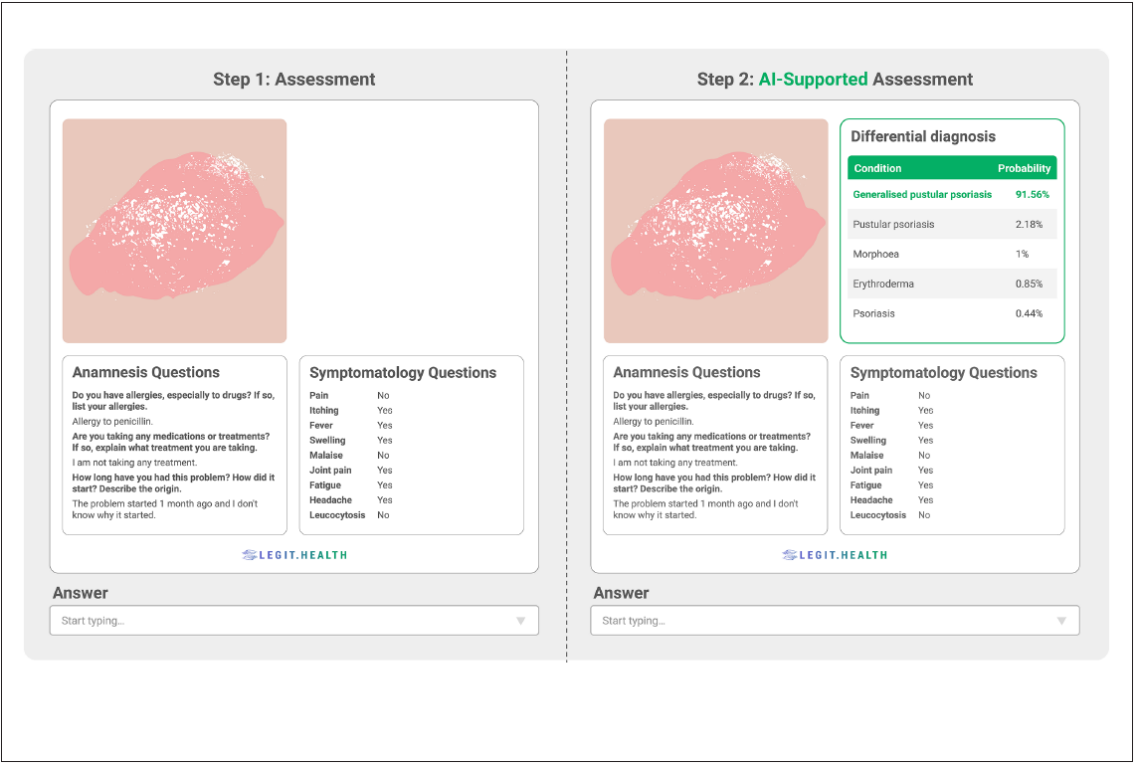
Comparison between the first and second stages of clinical evaluation. AI: artificial intelligence.

The multireader, multicase clinical evaluation, which took place virtually, consisted of reviewing 100 high-resolution images of 15 various skin conditions, including GPP and other visually similar dermatologic conditions. All participants were presented with the same dataset from the combined image pool. Each image was accompanied by a detailed patient anamnesis, including medical history, systemic symptoms, and any other relevant laboratory findings. This information was intended to mimic real-world clinical scenarios, providing a holistic context for the diagnosis. Each case was structured as a 2-stage sequence (Figure 2) to measure the impact of the Legit.Health medical device on the accuracy of HCPs in diagnosing GPP and other conditions.

In the first stage, HCPs were presented with the image and clinical information and recorded their initial diagnosis without assistance from the medical device. This first stage aimed to capture the baseline diagnostic accuracy of the HCP.

The second stage aimed to evaluate how the integration of Legit.Health aided the diagnostic process and whether it improved the identification of GPP by HCPs. The image was supplemented with the medical device’s top 5 diagnostic predictions, along with the corresponding confidence values for each prediction. These predictions were intended to serve as a second opinion, aiding the HCPs in refining their initial diagnoses. The HCPs were encouraged to consider the medical device’s output during their final diagnostic decisions, using its insights to enhance the accuracy and confidence of their initial clinical assessments.

In both stages, participants had to make their choices from a list of 406 conditions, which could be easily explored by typing in either English or Spanish. This list, which was curated from Legit.Health’s internal dataset, covered all pathologies encompassed by the device at different levels of detail, as well as those used in this study. Once all responses were collected, they were normalized to avoid redundant or synonymous terms.

Image quality may impact the effectiveness of the Legit.Health medical device and is a major concern both in clinical practice and in research. If the user inputs an image into the device that does not capture the region of interest correctly or that does not provide enough resolution to correctly identify the condition based on the pixels in the image, the reliability of the output may decrease. To overcome this limitation in this study, we used a specialized neural network for Dermatology Image Quality Assessment (DIQA), which was provided by Legit.Health [23]. All images included in this clinical evaluation were subjected to DIQA and successfully met the image quality requirements.

The participants first accessed the platform on June 24, 2024, on their computers. The first answer was recorded on June 25, 2024, and the last answer was recorded on July 6, 2024.

### Ethical Considerations

This study analyzed patient images from the EFFISAYIL 1 (NCT03782792) and EFFISAYIL 2 (NCT04399837) clinical trials, which were conducted in accordance with the trial protocols, the International Council for Harmonisation of Technical Requirements for Pharmaceuticals for Human Use good clinical practice guidelines, Regulation 536/2014 (European Union), the Japanese good clinical practice regulations, and applicable local regulations. The studies were approved by the ethics committees of participating institutions and countries. The study protocols were approved at each study site and/or country.

All patients from the EFFISAYIL 1 and EFFISAYIL 2 trials provided written informed consent, and confidentiality agreements were in place between the authors and Boehringer Ingelheim. Patient consent was obtained for the inclusion of patient photographs in this publication. More detailed information regarding data compilation, sharing policies, review board approvals, and ethical standards can be found in the original source publications for these trials [24,25].

The rest of the images were extracted from public and open dermatology atlases and open web images. Due to the retrospective use of anonymized, open-source data, no separate approval was required from an ethics committee. This study did not require ethics committee approval because it is observational and noninterventional.

The original protocols for the Effisayil 1 (NCT03782792) and Effisayil 2 (NCT04399837) trials explicitly allow for the sharing of deidentified participant-level data and clinical study documents for secondary analyses. The data-sharing process ensures that all shared materials are deidentified to protect participant privacy and remain within the scope of the original informed consent. Access to the clinical study data and images was obtained in accordance with the sponsor's (Boehringer Ingelheim) Transparency and Publication Policy. This access is granted to external authors to fulfill their roles under ICMJE criteria and is governed by a formal Legal Agreement that authorizes the secondary use of the data for this manuscript.

## Results

### Device Performance

The Legit.Health medical device achieved top-1, top-3, and top-5 sensitivity values of 0.80, 0.86, and 0.90, respectively, with specificity values exceeding 0.90 (Table 2), indicating robust performance across various diagnostic thresholds.

**Table 2.** Generalized pustular psoriasis (GPP) detection performance of Legit.Health before and after fine-tuning with additional GPP images.

Metric	Before fine-tuning	After fine-tuning
Top-1 sensitivity	0.4000	0.8026
Top-1 specificity	1.0000	0.9983
Top-3 sensitivity	0.8000	0.8633
Top-3 specificity	0.9994	0.9957
Top-5 sensitivity	0.8000	0.9002
Top-5 specificity	0.9986	0.9611

To better understand the medical device’s performance, we inspected the GPP images from the test set when another condition was predicted (top 1 prediction). The top 5 conditions with which GPP was most confused are shown in Table 3.

**Table 3.** Summary of the classes predicted in the top-1 position instead of generalized pustular psoriasis (GPP) assessed by Legit.Health.

Top 1 predicted condition	*ICD-11*^a^ code [21]	Number of misclassified GPP images
Generalized eczematous dermatitis of unspecified type	EA89	16
Pustular psoriasis^b^	EA90.4	15
Nonspecific lesion (no condition)	—^c^	12
Plaque psoriasis	EA90.0	12
Morphea	EB61	5

a *ICD-11*: *International Classification of Diseases, 11th Revision*.

b Pustular psoriasis (EA90.4) encompasses a set of images that include localized pustular psoriasis subcategories such as palmoplantar pustulosis (EA90.42); other specified pustular psoriasis (EA90.4Y); and pustular psoriasis, unspecified (EA90.4Z). GPP (EA90.40) was excluded from the pustular psoriasis group and included as an independent category.

c Not applicable.

### Clinical Evaluation

The overall diagnostic accuracy of HCPs across several different dermatologic conditions improved significantly with the use of Legit.Health. PCPs exhibited an increase in diagnostic accuracy of 17.74%, whereas dermatologists exhibited an improvement of 8.4%. Dermatologists changed their diagnoses more often than PCPs; on average, the switch ratio (ie, how many times a participant changed their responses) for dermatologists was 0.87 (SD 0.04), whereas for PCPs, it was 0.67 (SD 0.13). The overall switch ratio considering all HCPs was 0.72 (SD 0.14). The overall impact of the device was assessed using a McNemar test (Table 4), with the conclusion that the device had an impact on the improvement in diagnostic capabilities (*P<*.001).

**Table 4.** Results of the McNemar tests for all groups. The resulting contingency tables from these McNemar tests have been reorganized to represent positive rate (correct answer after device use), neutral positive rate (correct answer before and after use of the device), neutral negative rate (incorrect answer before and after use of the device), and negative rate (incorrect answer after use of the device).

Group	Positive rate, % (n/N)	Neutral positive rate, % (n/N)	Neutral negative rate, % (n/N)	Negative rate, % (n/N)
HCPs^a^	16.76 (236/1408)	46.31 (652/1408)	35.3 (497/1408)	1.63 (23/1408)
Primary care practitioners	19.61 (199/1015)	42.46 (431/1015)	36.06 (366/1015)	1.87 (19/1015)
Dermatologists	9.41 (37/393)	56.23 (221/393)	33.33 (131/393)	1.02 (4/393)

a HCP: health care practitioner.

To better understand the influence of the device outputs on the participants’ responses, we counted the number of diagnosis changes across several top-1 probability ranges and the position of the correct diagnosis. The motivation behind using only the top-1 probability is that it serves as a simple proxy for the system’s overall certainty for a given image. The results, presented in Table 5 & Table 6, show that participants who changed their diagnoses did so regardless of the device’s confidence or the position of the confirmed, ground-truth diagnosis.

**Table 5.** Influence of the device top-1 output and the ratio of changed diagnoses.

Device top-1 confidence	Number of images in range	Switch ratio
0.0‐0.2	5	0.63
0.2‐0.4	5	0.60
0.4-0-6	4	0.80
0.6‐0.8	17	0.71
0.8‐1.0	69	0.75

**Table 6.** Influence of the position of the correct condition on the ratio of changed diagnoses.

Position of correct condition	Number of images	Switch ratio
Top 1	76	0.67
Top 2	8	0.74
Top 3	2	0.71
Top 4	2	0.70
Top 5	1	0.77
Not in top 5	11	0.87

**Figure 3.**
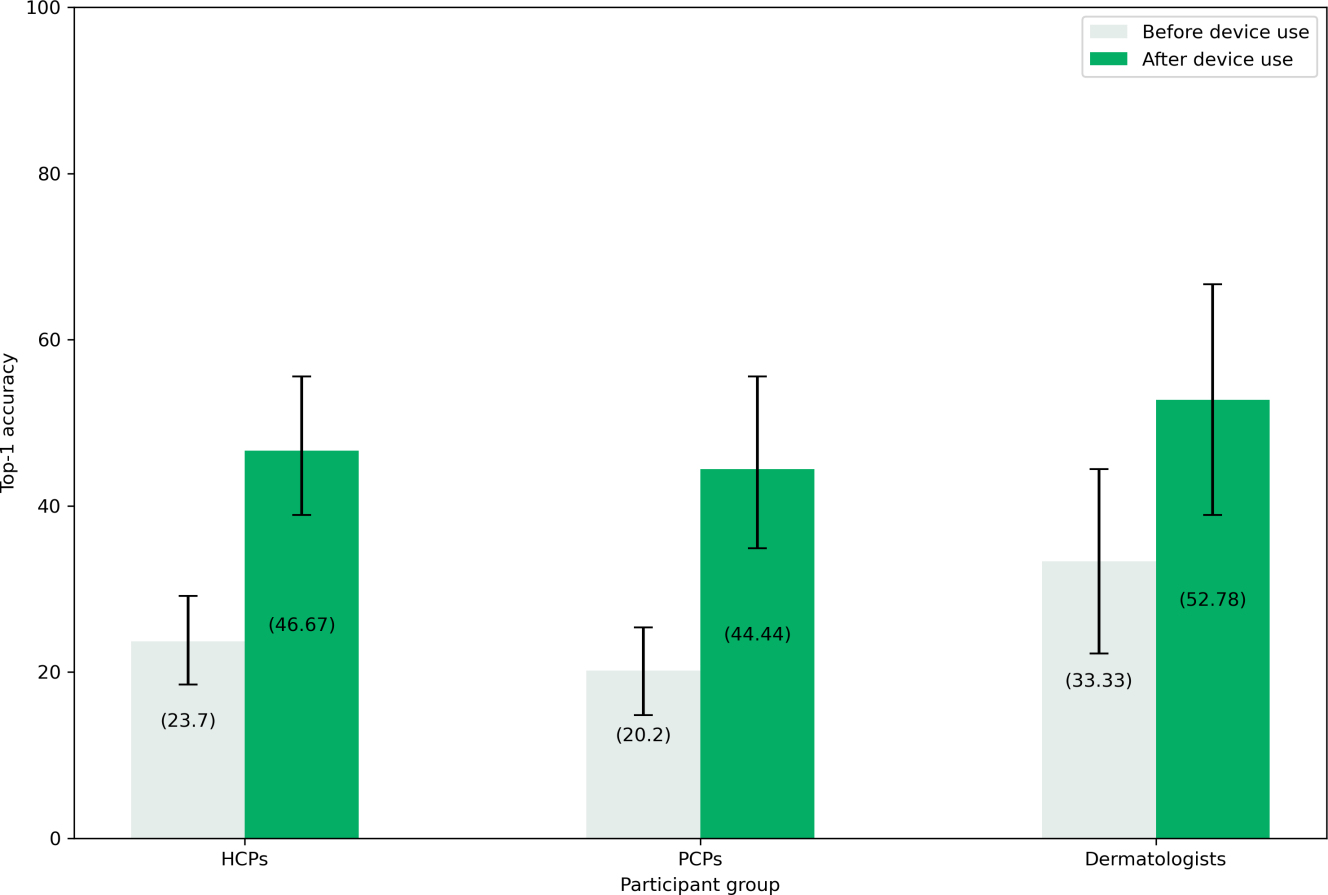
Top-1 accuracy of participants for generalized pustular psoriasis diagnosis before and after use of the device (with 95% CIs). HCP: health care practitioner; PCP: primary care practitioner.

The impact that Legit.Health had on diagnostic performance varied across conditions. For GPP (Figure 3), the diagnostic accuracy of HCPs increased from 23.7% to 46.7%, representing a 22.97% increase (Table 7). PCPs experienced a 24.24% increase in diagnostic accuracy (Table 8), whereas dermatologists experienced a 19.45% increase (Table 9). For HS, PCPs showed a 10.11% increase in diagnostic accuracy, with no substantial change observed for dermatologists (2.85%). One of the most notable improvements was observed in the diagnosis of palmoplantar pustulosis, where PCPs exhibited a 47.82% increase in diagnostic accuracy. Overall, Legit.Health enhanced diagnostic performance in 16.76% of cases, demonstrating significant diagnostic improvements (*P*<.001) for dermatologic conditions that are frequently misdiagnosed due to similar clinical presentations.

**Table 7.** Top-1 accuracy of all participants.

Condition	*ICD-11*^a^ code	Accuracy before use of the device (%; 95% CI)	Accuracy after use of the device (%; 95% CI)	Difference (%; 95% CI)
Acne	ED80	37.50 (29.27-46.15)	56.25 (47.06-66.67)	18.75 (8.50-29.41)
Acne conglobata	ED80.41	18.40 (12.31-23.81)	38.40 (27.03-50.00)	20.00 (13.52-27.42)
Drug-induced acute generalized exanthematous pustulosis	EH67.0	5.00 (0.00-9.38)	5.00 (0.00-9.38)	0.00 (0.00-0.00)
Generalized eczematous dermatitis of unspecified type	EA89	71.34 (66.66-76.14)	73.17 (68.33-78.41)	1.83 (0.00-3.90)
Generalized pustular psoriasis	EA90.40	23.70 (18.52-29.17)	46.67 (38.89-55.56)	22.97 (18.52-28.39)
Hidradenitis suppurativa	ED92.0	85.48 (79.71-92.00)	93.55 (89.87-98.44)	8.07 (3.79-12.65)
Impetigo	1B72	57.43 (47.96-67.36)	76.35 (66.25-88.78)	18.92 (12.86-25.00)
Palmoplantar pustulosis	EA90.42	45.31 (30.56-56.76)	79.69 (71.43-88.89)	34.38 (22.58-47.37)
Pemphigus vulgaris	EB40.0	28.77 (18.17-40.00)	56.16 (40.00-74.42)	27.39 (16.00-39.40)
Plaque psoriasis	EA90.0	91.89 (87.18-97.50)	97.30 (94.87-100.00)	5.41 (2.04-9.09)
Seborrheic dermatitis and related conditions	EA81.Z	75.34 (68.42-82.86)	90.41 (82.86-100.00)	15.07 (9.99-20.93)
Seborrheic keratosis	2F21.0	94.67 (91.11-100.00)	97.33 (95.00-100.00)	2.66 (0.00-5.00)
Severe inflammatory acne	ED80.4Z	10.61 (4.88-16.13)	43.94 (33.33-53.85)	33.33 (26.19-41.38)
Subcorneal pustular dermatosis	EB2Y	2.67 (0.00-5.00)	2.67 (0.00-5.00)	0.00 (0.00-0.00)
Tinea corporis	1F28.Y	36.36 (28.85-42.60)	62.50 (55.00-71.15)	26.14 (17.28-34.80)

a *ICD-11*: *International Classification of Diseases, 11th Revision*.

**Table 8.** Top-1 accuracy of primary care practitioners.

Condition	*ICD-11*^a^ code	Accuracy before use of the device (%; 95% CI)	Accuracy after use of the device (%; 95% CI)	Difference (%; 95% CI)
Acne	ED80	36.36 (25.93-46.89)	61.36 (50.00-73.91)	25.00 (10.81-40.93)
Acne conglobata	ED80.41	21.18 (13.46-28.89)	48.24 (35.19-62.97)	27.06 (20.00-35.71)
Drug-induced acute generalized exanthematous pustulosis	EH67.0	0.00 (0.00-0.00)	0.00 (0.00-0.00)	0.00 (0.00-0.00)
Generalized eczematous dermatitis of unspecified type	EA89	68.33 (63.08-73.85)	70.83 (64.47-77.27)	2.50 (0.00-5.26)
Generalized pustular psoriasis	EA90.40	20.20 (14.81-25.40)	44.44 (34.92-55.56)	24.24 (18.50-31.48)
Hidradenitis suppurativa	ED92.0	82.02 (74.55-90.74)	92.13 (87.04-100.00)	10.11 (5.00-15.38)
Impetigo	1B72	50.00 (39.71-60.29)	72.22 (58.57-87.94)	22.22 (15.00-30.89)
Palmoplantar pustulosis	EA90.42	32.61 (23.08-41.94)	80.43 (74.07-86.21)	47.82 (37.93-58.35)
Pemphigus vulgaris	EB40.0	22.64 (10.53-36.36)	43.40 (22.86-66.67)	20.76 (8.58-33.38)
Plaque psoriasis	EA90.0	88.89 (82.76-94.87)	96.30 (92.00-100.00)	7.41 (2.86-11.77)
Seborrheic dermatitis and related conditions	EA81.Z	69.81 (60.71-78.79)	88.68 (78.57-100.00)	18.87 (12.12-26.09)
Seborrheic keratosis	2F21.0	92.73 (86.67-100.00)	96.36 (92.00-100.00)	3.63 (0.00-6.67)
Severe inflammatory acne	ED80.4Z	10.87 (3.85-17.24)	50.00 (37.92-62.96)	39.13 (30.77-48.39)
Subcorneal pustular dermatosis	EB2Y	0.00 (0.00-0.00)	0.00 (0.00-0.00)	0.00 (0.00-0.00)
Tinea corporis	1F28.Y	29.69 (23.53-35.29)	59.38 (50.00-70.59)	29.69 (19.04-42.50)

a *ICD-11*: *International Classification of Diseases, 11th Revision*.

**Table 9.** Top-1 accuracy of dermatologists.

Condition	*ICD-11*^a^ code	Accuracy before use of the device (%; 95% CI)	Accuracy after use of the device (%; 95% CI)	Difference (%; 95% CI)
Acne	ED80	40.00 (30.00-50.00)	45.00 (30.00-60.00)	5.00 (0.00-10.00)
Acne conglobata	ED80.41	12.50 (5.00-20.00)	17.50 (10.00-25.00)	5.00 (0.00-10.00)
Drug-induced acute generalized exanthematous pustulosis	EH67.0	18.75 (0.00-37.50)	18.75 (0.00-37.50)	0.00 (0.00-0.00)
Generalized eczematous dermatitis of unspecified type	EA89	79.55 (72.73-86.36)	79.55 (72.73-86.36)	0.00 (0.00-0.00)
Generalized pustular psoriasis	EA90.40	33.33 (22.22-44.44)	52.78 (38.89-66.67)	19.45 (16.67-22.23)
Hidradenitis suppurativa	ED92.0	94.29 (86.67-100.00)	97.14 (93.33-100.00)	2.85 (0.00-6.67)
Impetigo	1B72	77.50 (65.00-90.00)	87.50 (75.00-100.00)	10.00 (5.00-15.00)
Palmoplantar pustulosis	EA90.42	77.78 (50.00-100.00)	77.78 (50.00-100.00)	0.00 (0.00-12.50)
Pemphigus vulgaris	EB40.0	45.00 (30.00-60.00)	90.00 (80.00-100.00)	45.00 (30.00-60.00)
Plaque psoriasis	EA90.0	100.00 (100.00-100.00)	100.00 (100.00-100.00)	0.00 (0.00-0.00)
Seborrheic dermatitis and related conditions	EA81.Z	90.00 (80.00-100.00)	95.00 (90.00-100.00)	5.00 (0.00-10.00)
Seborrheic keratosis	2F21.0	100.00 (100.00-100.00)	100.00 (100.00-100.00)	0.00 (0.00-0.00)
Severe inflammatory acne	ED80.4Z	10.00 (0.00-20.00)	30.00 (20.00-40.00)	20.00 (10.00-30.00)
Subcorneal pustular dermatosis	EB2Y	10.00 (0.00-20.00)	10.00 (0.00-20.00)	0.00 (0.00-0.00)
Tinea corporis	1F28.Y	54.17 (41.67-66.67)	70.83 (58.33-83.33)	16.66 (8.33-25.00)

a *ICD-11*: *International Classification of Diseases, 11th Revision*.

## Discussion

### Principal Findings

In this study, the Legit.Health medical device enhanced HCPs’ diagnostic accuracy for GPP, especially among PCPs with limited specialized dermatologic expertise. Legit.Health demonstrated its potential in helping HCPs distinguish GPP from other dermatologic conditions, which could fulfill a critical need for timely and precise diagnosis of this rare and complex disease. By enhancing diagnostic accuracy, AI-powered tools such as Legit.Health have the potential to reduce health care disparities, particularly in underserved areas with limited access to dermatologic specialists.

While individual neural networks for disease recognition have previously been shown to be useful for the automatic assessment of the severity of HS [17], urticaria [18], or atopic dermatitis [19] cases, with results comparable with those of human experts, this clinical evaluation is the first to reveal substantial improvements in diagnostic accuracy across various skin conditions, highlighting the potential of this medical device in reducing diagnostic errors; streamlining clinical decision-making; and, ultimately, improving patient outcomes.

These findings highlight the critical role that AI can play in bridging the expertise gap in dermatology, particularly for rare and complex diseases such as GPP. By aiding HCPs in differentiating GPP from other skin conditions, the Legit.Health medical device could facilitate timely and accurate diagnoses, which are crucial for prompt and effective treatment. Improved diagnostic accuracy can, in turn, reduce the risk of complications and improve quality of life for patients with GPP. This capability could be especially valuable in primary care environments, where immediate access to dermatologic specialists may not be possible.

Moreover, the performance of Legit.Health across a range of conditions highlights its versatility, further validating its utility and potential to improve diagnostic accuracy in the broad field of dermatology. Thanks to the extensive dataset used to train the algorithm, the device can provide the practitioner with a broader scope of pathologies, making them aware of less common diseases. We believe this could have occurred to some extent in this study: the participants may be biased toward common diseases (ie, the ones they are more likely to encounter in real life), and the output of the device changed their final assessments to more specific diagnoses (such as GPP or HS). Our results support the hypothesis that the device outputs served as a trigger to reconsider the diagnosis rather than an authoritative answer: the mere presence of an alternative diagnosis was enough to make the participants switch regardless of the confidence metric attached to it.

### Limitations

However, this study has several limitations. First, this study only analyzed the impact of 1 medical device. However, Legit.Health was deemed appropriate for this study due to its approved regulatory status and previous published evidence supporting its use in clinical assessments [17-19,26]. Furthermore, to the best of our knowledge, there is no other equivalent device with GPP diagnosis capabilities. For that reason, the results of this study may not generalize to other similar technologies developed for the assessment of different conditions. Further studies may focus on performing a similar assessment using different devices. Another limitation of this study is that, due to the rarity of GPP, assembling a diverse dataset is particularly challenging. Higher skin phototypes (ie, darker skin) [27] were underrepresented in the GPP images used to train the algorithm; this limitation is planned to be addressed in future iterations by supplementing the dataset with additional images with darker skin tones.

This study has demonstrated that the Legit.Health device had a positive impact on participants’ performance. However, our analysis of users’ dynamics before and after use of the device was limited by the amount of data collected during the experiment. In future work, additional variables such as seniority and the participants’ perceived confidence in their initial responses should also be taken into account.

It is also worth noting that image-based diagnostic tools face limitations beyond image quality alone. The use of DIQA assured that there were no low-quality images, but there can be other issues that may impact the performance of the medical device. During the study, an interesting pattern of confusion between GPP and “nonspecific lesions” was observed, often in images with subtle or low-intensity visible signs. This highlights the crucial role of users in capturing high-quality images with a clear focus on the region of interest.

Furthermore, a noted limitation of this study is that it only considered teledermatology assessment based on images and information transmitted electronically. This context may not be fully replicable in in-person clinical environments where HCPs have the advantage of direct patient examination, additional questioning, and follow-up assessments. Nevertheless, this study incorporated detailed anamnesis and other relevant clinical information alongside the images to provide a comprehensive evaluation and equate the remote consultation with an in-person consultation.

Overall, this study shows that the use of an AI-powered medical device such as Legit.Health represents a potential advancement in dermatologic diagnostics. Legit.Health enhanced the accuracy of GPP diagnoses and could provide valuable support to HCPs, which in turn may improve patient care and outcomes.

### Conclusions

Use of Legit.Health was associated with an improvement in the accuracy of HCPs in diagnosing GPP and other dermatologic conditions such as HS. This AI-powered medical device could be particularly beneficial in primary care settings, where specialized dermatologic expertise may not be available. This study revealed that the implementation of Legit.Health enhanced the overall diagnostic accuracy of HCPs and reduced the rate of misdiagnoses, potentially streamlining the decision-making process.

Although this study only considered the medical device Legit.Health, these results highlight the promise of the growing field of AI technologies as a beneficial tool for the future of dermatologic diagnostics and patient management.

## Abbreviations

AI: artificial intelligence
CLEAR Derm: Checklist for Evaluation of Image-Based Artificial Intelligence Reports in Dermatology
DIQA: Dermatology Image Quality Assessment
GPP: generalized pustular psoriasis
HCP: health care practitioner
HS: hidradenitis suppurativa
*ICD-11*: *International Classification of Diseases, 11th Revision*
PCP: primary care practitioner
